# Smartphone Determination of Formaldehyde in Milk and Cosmetics Samples Using Gas-Diffusion Microextraction Devices Prepared by 3D Printing

**DOI:** 10.1021/acsomega.6c03601

**Published:** 2026-07-02

**Authors:** Juliana Casanova Pinho, Gabriel Baroffaldi Piassalonga, Saidy Cristina Ayala-Durán, João Pedro Silva, Josias Merib, Jared L. Anderson, Maria Valnice Boldrin Zanoni, Paulo Clairmont Feitosa de Lima Gomes

**Affiliations:** † Sao Paulo State University (UNESP), Institute of Chemistry, Department of Analytical Chemistry, Physical Chemistry and Inorganic Chemistry, National Institute for Alternative Technologies of Detection, Toxicological Evaluation, and Removal of Micropollutants and Radioactives (INCT-DATREM), São Paulo State University (UNESP), Araraquara, São Paulo 14800-060, Brazil; ‡ 117303 Universidade Federal de Ciências da Saúde de Porto Alegre, Porto Alegre, Rio Grande do Sul, Brasil; Programa de Pós-graduação em Biociências e Departamento de Farmacociências, Porto Alegre, Rio Grande do Sul 90050-170, Brazil; § Department of Chemistry, 1177 Iowa State University, Ames, Iowa 50011, United States

## Abstract

This study reports the development of a low-cost direct method for determining formaldehyde in milk and cosmetic samples. Formaldehyde was extracted from the samples using a 3D-printed gas-diffusion microextraction module, and detection was performed with a smartphone coupled to the module for colorimetric analysis. An algorithm was created in MATLAB to automate data processing. Formaldehyde was extracted from milk by acidifying the solution and exploiting the salting-out effect. Analytical curves for different types of milk (whole, skim, semiskim, lactose-free, and ultrahigh-temperature-processed) were generated using smartphone-based detection and compared with those obtained using a traditional UV/Vis spectrophotometer. A cosmetic sample used in this research was a straightening hair cream, quantified using the standard addition calibration method. The formaldehyde concentration in the straightening hair cream was determined from smartphone images to be 0.149% (w/w), whereas the spectrophotometric method yielded 0.144% (w/w). To evaluate the method’s accuracy, a headspace gas chromatography–mass spectrometry (HS-GC-MS) method was developed and applied to quantify the samples using the standard addition method. The accuracy of the proposed method exceeded 90% when compared with digital image data obtained using chromatographic and spectrophotometric methods.

## Graphical abstract

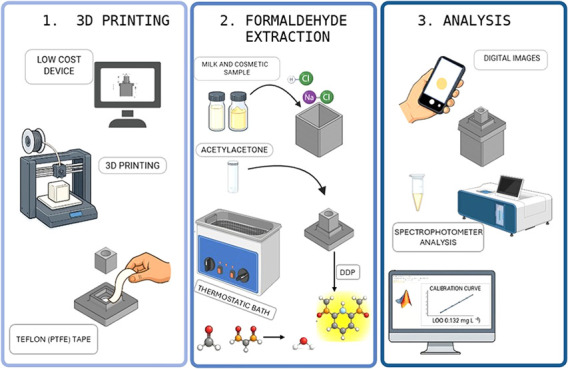

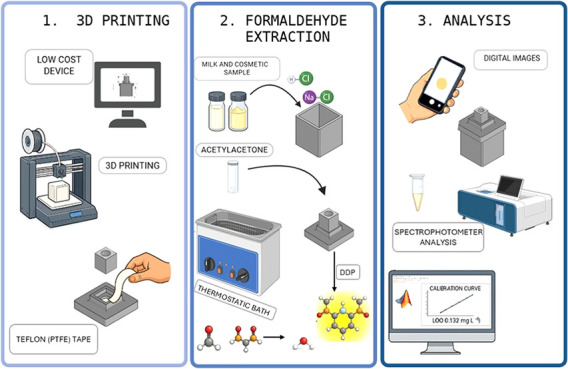

## 1 Introduction

Formaldehyde is a well-known compound for its antimicrobial activity and toxicity. The International Agency for Research on Cancer (IARC) classifies formaldehyde as carcinogenic to humans.^1^ In foods of animal and plant origin, formaldehyde occurs naturally through the oxidation of methanol.^2^ In vegetables, the main source of methanol is pectin,^3^ while in smaller quantities, it is generated through the demethylation of DNA, RNA, and proteins.^2^ In animals (cattle and poultry), methanol is produced by the demethylation of DNA, RNA, histones, and pectin contained in the vegetables in their diet.^2^ Formaldehyde can be generated directly by the deamination of methylamine, oxidation of glycine, and the glycine cleavage system.^4^ Normal levels of formaldehyde vary from 1.00 ^5^ to 493 mg kg^–1^.^6^ Industrial food processing can generate formaldehyde using carbohydrates, lipids, ascorbic acid, and amino acids as precursors.^2^

Formaldehyde can be used as a food adulterant because its antimicrobial properties help extend shelf life. In the European Union, Regulation (EC) No. 1333/2008 lists the additives approved for food use, excluding formaldehyde,^7^ but it was permitted in milk intended for pig feed until 2018.^8^ Adulteration of milk with formaldehyde remains a significant issue in developing countries. Evidence of this practice has been found in commercially available milk in several nations, including India (2024),^9^ and Pakistan (2025).^10^ Milk analysis in Brazil uses method B of AOAC 931.08, which does not require quantitative testing.^11–13^ In the United States, formaldehyde analysis is not mentioned in the United States Public Health Service (USPHS) milk sanitation program, as outlined in the Pasteurized Milk Ordinance guide.

Another use for formaldehyde is in the cosmetics industry, where it is used in water-based products as a preservative to prevent the growth of microbial life,^14^ thereby extending the product’s shelf life. Brazilian legislation allows the use of formaldehyde in personal hygiene products.^15^ In hygiene products, it is permitted as a preservative, and in nail polishes, it serves as a nail hardener. Product labels must only contain formaldehyde in concentrations greater than 0.05%, and its use in aerosols and sprays is prohibited.

The cosmetics industry uses formaldehyde-releasing compounds to preserve its water-based products. Compounds called “formaldehyde releasers” enable the slow release of formaldehyde through several consecutive hydrolysis reactions of these compounds in contact with water. Well-known examples are quaternium-15, imidazolidinyl urea, diazolidinyl urea, 1,3-Bis­(hydroxymethyl)-5,5-dimethylimidazolidine-2,4-dione (DMDM hydantoin) and 2-bromo2-nitropropane-1,3-diol.^16^ European legislation allows the use of formaldehyde in cosmetics up to 0.2%, and formaldehyde releasers up to 0.6%.^17^ Brazilian legislation follows the same directives as the European Union regarding formaldehyde, but does not mention formaldehyde releasers.^15^

Several colorimetric methods have been developed to determine formaldehyde. The Nash method uses acetylacetone in an ammonium acetate/acetic acid buffer to produce a yellow compound, which can also be quantified by fluorescence or absorbance.^18,19^ The presence of acetaldehyde, amines, formaldehyde polymers, periodate, and sulfites can cause interferences.^20^ Other methods used for determination contain larger amounts of interferents, such as 2,4-dinitrophenylhydrazine, which reacts with carbonyl compounds,^21^ and chromotropic acid + sulfuric acid, which reacts with other aldehydes.^22^ Chromatographic approaches can also be used to determine formaldehyde, including GC-FID,^23^ GC-MS,^24^ and HPLC.^25^ While these methods offer high precision, their requirement for high-cost instrumentation and specialized users makes them less accessible for effective quality monitoring in developing countries.

To overcome these limitations, researchers at Porto University have developed more accessible, cost-effective techniques for sample preparation and analysis. Gas-diffusion microextraction (GDME) is a simple, low-cost technique for analyzing volatile and semivolatile compounds. It also enables the isolation, concentration, and derivatization of analytes in a single, rapid step, as it does not require extensive sample preparation.^26^ The device was developed and patented in,^27^ allowing for the analysis of liquid and solid samples. The versatility of this technique enables the analysis of solid samples without prior pretreatment. When a derivatization reaction is performed, it is possible to improve both extraction efficiency and selectivity^28^ The main components of this extraction system are a Falcon tube, a thermostatic bath, and an extractor module.^29^ The extractor module is made of small-sized Teflon (also called PTFE) and features a microporous membrane inside the device, to which the derivatizing reagent is added. The membrane prevents solvent diffusion but allows for the mass transfer of volatile and semivolatile analytes. The module is inserted into a Falcon tube containing the sample and is thermally controlled. Over time, the analyte in the sample diffuses and meets the reagent previously placed on top of the membrane, where it is extracted from the sample and preconcentrated in the derivatizing solution. When combined with gas-diffusion microextraction, derivatization can be more effective by increasing the amount of analyte collected and facilitating the analysis. Ferreira demonstrated the use of this extraction technique in his work with solid samples,^29^ showing it to be a good platform for sampling volatile compounds in bread samples (butane-2,3-dione, pentane-2,3-dione, and hexane-2,3-dione)^30^ and monitoring simple aldehydes (formaldehyde, acetaldehyde, furfural, propanal, butanal, benzaldehyde) during the production process of a cork stopper.^28,31^ This technique can also be applied to liquid samples; Goncalves et al. utilized the GDME device to analyze aldehydes in beer samples.^32^ Nevertheless, traditional GDME devices are typically designed to fit specific commercial containers, such as 50 mL Falcon tubes or Duran flasks. This dependency creates a geometric constraint, limiting the analysis to samples that accommodate these specific dimensions.

3D printing provides an avenue to minimize the project’s cost while making it more accessible, particularly in the manufacturing of GDME. This technique enables the creation of solid materials from a custom design, with advantages including low cost, ease of design, adaptability, and the ability to reproduce prototypes, making additive manufacturing a viable option for a user’s specific requirements.

Another significant environmental concern involves the extensive use of PTFE in the fabrication of traditional GDME devices. The manufacturing and processing of fluoropolymer components can release per- and polyfluoroalkyl substances (PFAS), which are classified as contaminants of emerging concern (CECs) due to their extreme persistence, often termed chemicals forever, and their tendency to bioaccumulate in aquatic ecosystems, posing endocrine and immunological risks to humans.^33^ In this context, reducing the reliance on high-mass PTFE components is a strategic step toward more sustainable analytical chemistry. While conventional devices typically require specialized PTFE filters that are costly (approximately USD 50.00) and weigh approximately 50.0 g, our design uses thin Teflon tape, which offers a dual advantage: it drastically lowers costs (to approximately USD 0.50) and significantly reduces the total mass of fluorinated material required (approximately 0.165 g used). This approach makes the device more accessible and environmentally friendly by employing readily available materials commonly used in sealing applications.

In this work, a novel method for determining formaldehyde in food and cosmetic samples is introduced. It utilizes a 3D-printed GDME device that enables formaldehyde analysis at a lower cost and in personalized sizes, tailored to the sample size and the amount required for extraction and quantification. Additionally, the study aims to achieve direct coupling of extraction and detection and to assess the reliability and versatility of using a smartphone-based detector in chemical analysis.

## 2 Materials and Methods

### 2.1 Chemicals and Reagents

All reagents used were analytical grade. Formaldehyde (37% w/w), hydrochloric acid (37% w/w), and NaCl (99% w/w) were purchased from NEON (Suzano - SP - Brazil). Acetylacetone (99% w/w) and ammonium acetate (98% m/m) were purchased from Merck (Jacarepaguá, RJ, Brazil). Black ABS filament was purchased from GTMax3D (Americana - SP - Brazil). SYLGARD 184 was purchased from DOWSIL (Midland - MI - USA). Water was produced in a laboratory using an ion-exchange resin, with a conductivity below 5.0 μS.

Formaldehyde solutions 1000 and 5000 mg L^–1^ were prepared every 2 weeks. Acetylacetone solution was prepared by weighing 3.85 g of ammonium acetate, 75 μL of glacial acetic acid, 50 μL of acetylacetone, and diluting with water in a 50 mL volumetric flask. HCl solution was prepared by diluting 1:1 with deionized water. Deionized water was used for all chemical analyses and the washing of glassware.

### 2.2 Milk and Straightening Hair Samples

The different types of milk samples (e.g., whole, semi-skim, skim, lactose-free, and pasteurized) were purchased from a supermarket in Araraquara, Brazil. Straightening cream was purchased from a local store in Araraquara, Brazil, specializing in beauty products. The samples were chosen because their labels stated “formaldehyde-free” but contained DMDM hydantoin, a common formaldehyde releaser used in the cosmetics industry.

### 2.3 Instrumentation

Spectrophotometric measurements were performed on a Bel UV-M51 spectrophotometer at a wavelength of 412 nm using quartz cuvettes with an optical path of 1 cm and a volume of 0.70 mL. Images were taken with an iPhone 11 smartphone. All 3D printed parts were designed in Autodesk Inventor, sliced with Ultimaker Cura, and produced on a GTMax3D Core A2v2 3D printer. Image processing was done using MATLAB. Gas chromatographic analyses were performed using a Shimadzu GC2010 plus with an automatic sampler AOC6000, coupled to a mass spectrometer QP-2020.

### 2.4 Gas-Diffusion Microextraction Design and Evaluation

GDMEs produced by FDM 3D printing feature small channels that allow formaldehyde to pass through, making their determination challenging. To cover these channels, a PDMS polymer was used at a 1:10 ratio of polymerizing agent to monomer. PDMS is a polymer that requires heating at 60 °C for 1 h. Therefore, the 3D-printed GDME was designed in a cubic shape to facilitate PDMS application and allow the polymer to form a uniform layer on each inner face of the GDME. Different GDME form factors were evaluated, with their height, width, and depth varied with the different dimensions shown in Table S1. Figure 1a represents prototype 2 of the printed GDME device, while Figure 1b,c show the assembly process of the GDME. The height of GDME devices was adjusted to maintain the same internal volume, ensuring the headspace volume remained constant and did not affect the extractions. A detailed illustration showcasing the distinct geometries and internal dimensions of all studied GDME models is provided in the Supporting Information (Figure S1). Prototype 6 was designed to evaluate the increase in headspace volume during extractions when compared to prototype 2. As a cheaper alternative to using the hydrophobic Mitex PTFE membrane, this work used PTFE plumbing tape. Two pieces of tape (18 mm width) were used and arranged perpendicularly to each other to cover the central hole.

**Figure 1:**
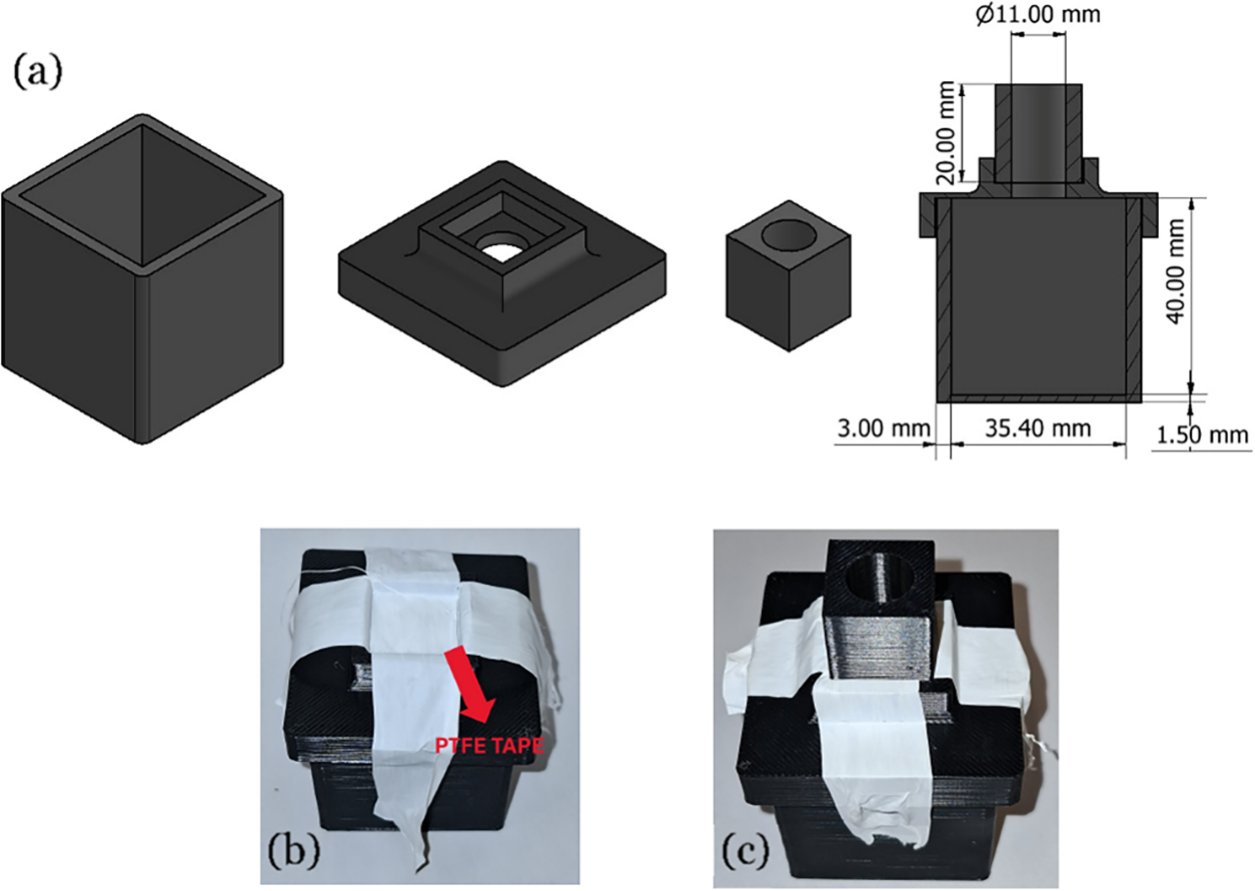
Technical drawings and dimensions of the 3D printed extraction system components: (a) GDME Prototype 2 illustration and detailed specifications. (b) GDME with the PTFE applied (c) final assembly of the GDME featuring PTFE tape and the support for the derivatizing solution.

Different GDME device designs were tested using formaldehyde solubilized in water. At the bottom of the GDME device, 25 mL of water and 125.0 μL of formaldehyde solution (5000 mg L^–1^) were added, and at the top of the hydrophobic membrane, 500.0 μL of acetylacetone solution was added. The GDME devices were placed in a water bath at 70 °C for 50 min. After the extraction was completed, a 10 min period was allowed for the absorbance to stabilize before it was measured by the spectrophotometer. The steps of the described procedure are detailed in Figure 2a.

**Figure 2:**
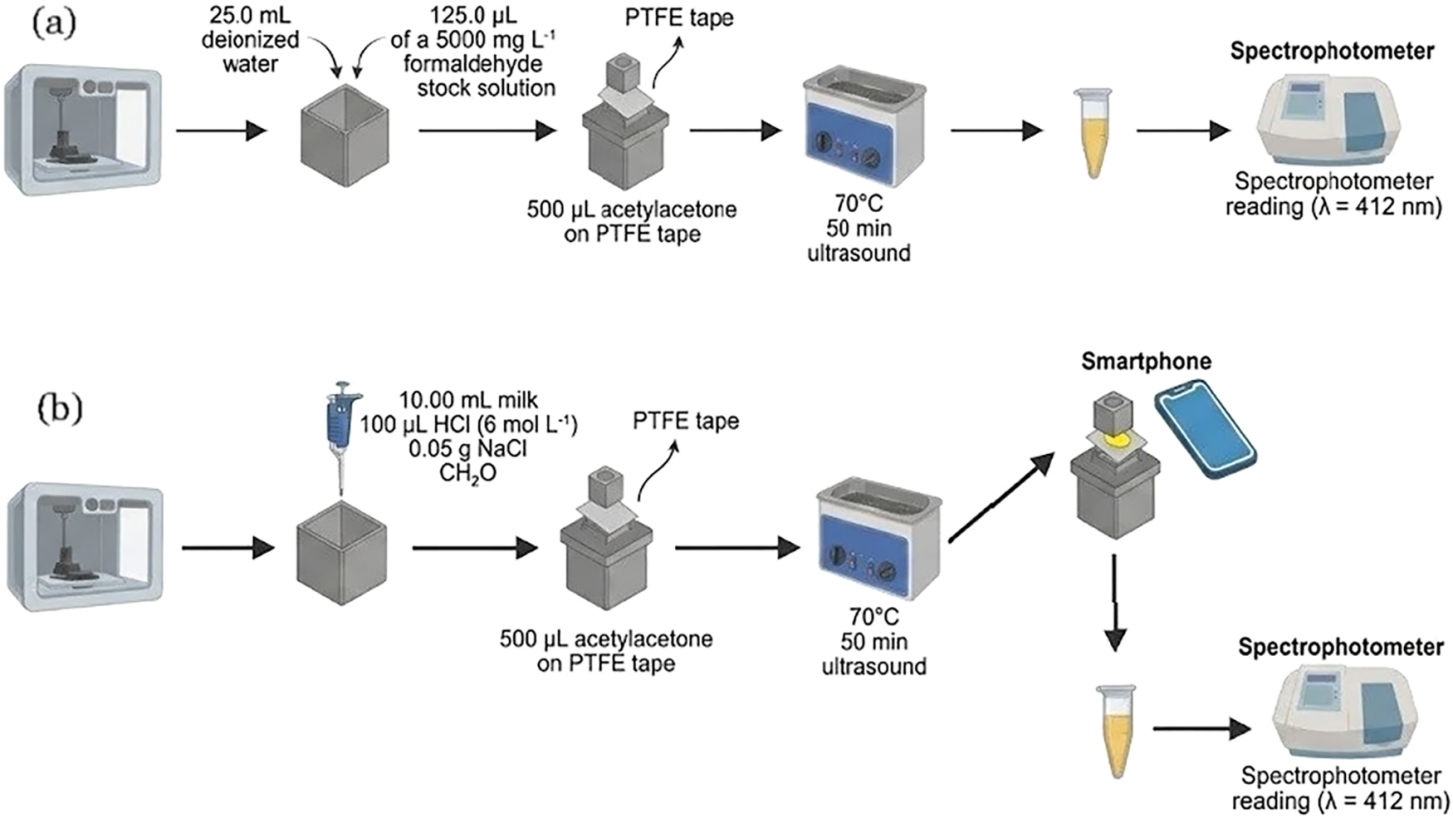
Schematic representation of the experimental workflows: (a) preliminary procedure for the GDME design evaluation, and (b) optimized analytical procedure for formaldehyde determination in milk samples.

### 2.5 Smartphone Detection and Image Processing

Formaldehyde and acetylacetone, in the presence of ammonia, react generating 3,5-diacetyl-1,4-dihydrolutidine (DDL), a yellow compound with maximum absorbance at 412 nm,^6,20^ absorbing in the violet/blue region of the electromagnetic spectrum. The selectivity of the colorimetric detection was also considered. The Hantzsch reaction is remarkably specific for formaldehyde, with interference from other aldehydes negligible even at concentrations up to 1000-fold higher than the analyte. This high selectivity, combined with the gas diffusion separation process provided by the GDME device—which prevents nonvolatile matrix components from reaching the donor solution—ensures that the absorbance measured at 412 nm is accurately attributed to the formation of the DDL derivative.^34,35^

To ensure uniformity across images, a support was designed to attach the smartphone to the GDME device (Figure 3a), while Figure 3b,c show the printed support. The camera parameters were adjusted to achieve a white color balance with the smartphone light and to focus on the derivatizing solution. The camera parameters were then fixed, and all analytical data were collected. The camera settings were as follows: resolution: 72 DPI; flash control: On; exposure adjustment: 0; lens aperture: f/1.8; focal length: 4.25 mm.

**Figure 3:**
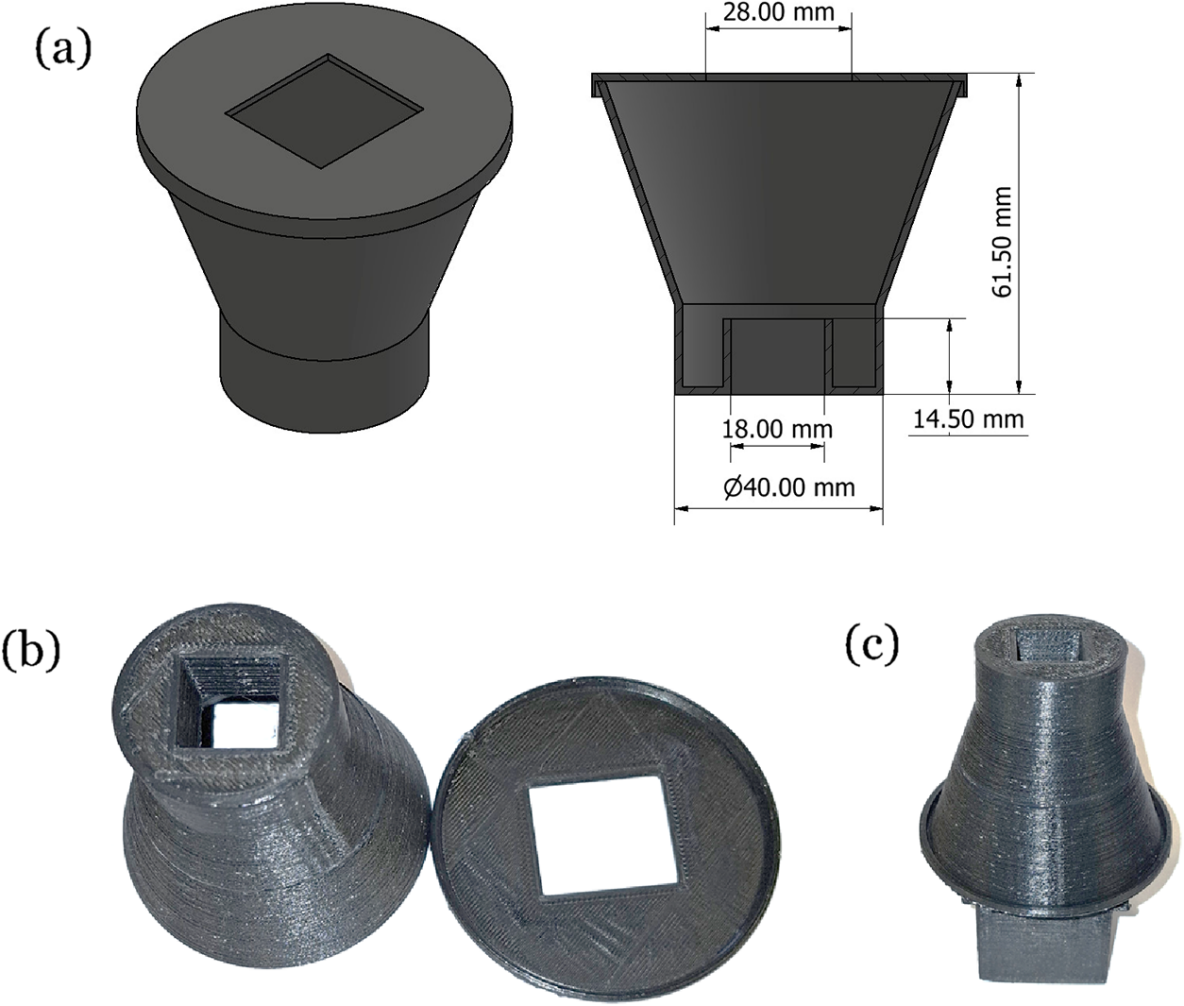
(a) Smartphone support design and dimensions. (b) 3D-printed smartphone support designed for attachment to the GDME; (c) fully coupled system showing the smartphone support attached to the GDME for image acquisition.

The images were processed using MATLAB. The images obtained by the smartphone (Figure 4a) showed saturated pixels from the flash and darker pixels corresponding to the GDME wall. To facilitate the analysis, a MATLAB algorithm was developed to automate image processing. The first step was to use the Color Thresholder app in the MATLAB application suite to generate code that filters the image for the lightest and darkest regions, as shown in Figure 4b. When the image was inserted into the Color Thresholder, different color spaces were enabled for processing the photographs: Red, Green, Blue (RGB); Hue, Saturation, and Value (HSV); Brightness, Blue-difference chroma, and Red-difference chroma (YCbCr); Lightness, Red-green axis, Yellow-blue axis (L*a*b*).^36^ The RGB space was chosen, allowing for the filtering of pixel values to be analyzed. Values ranging from 115 to 240 in the blue matrices were selected to create the analytical curve.

**Figure 4:**
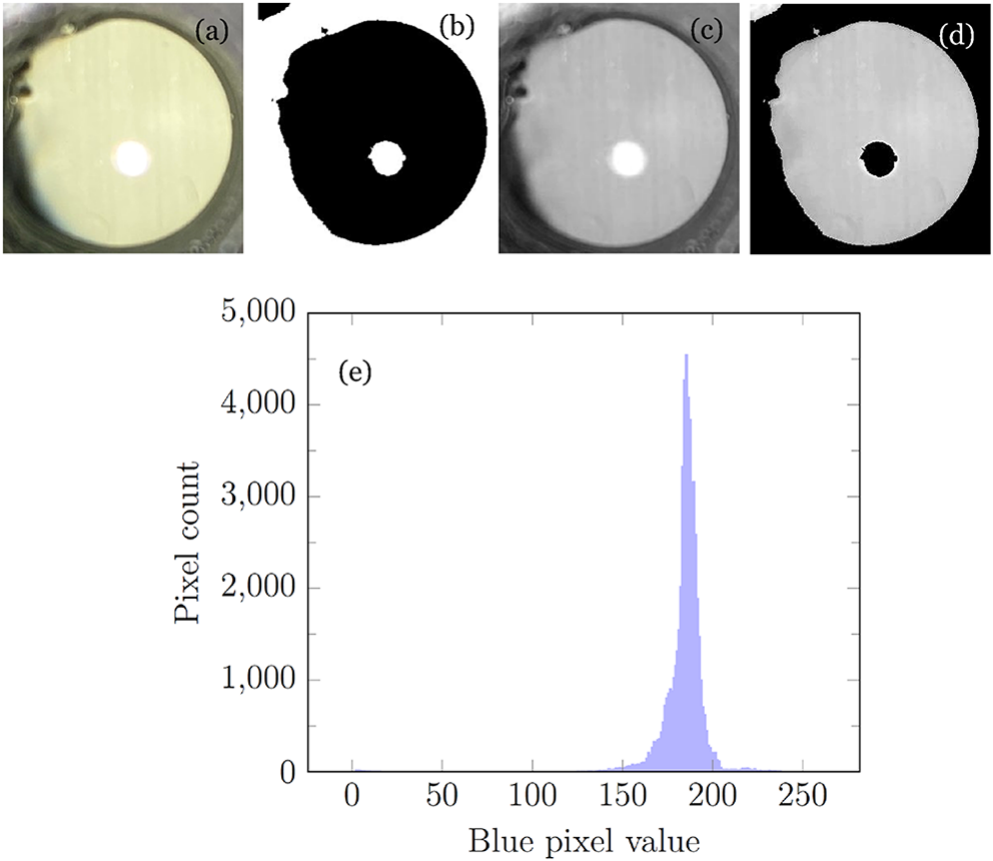
Step-by-step image processing. (a) Image obtained by smartphone. (b) Filter applied to the image, with the black region being used to construct the histogram. (c) Blue component of the image. (d) Filter applied to the blue matrix of the image. (e) Example of a histogram generated after filtering the blue component of the smartphone image.

Using the chosen values, the Color Thresholder application generated MATLAB code that was inserted into the Image Batch Processor, and individual filters were generated for each analyzed image. The next step was to select the blue matrix from the digital images (Figure 4c) and then subtract the filter (see Figure 4d). Finally, a histogram, shown in Figure 4e, was generated to determine the value of the pixels with the largest population. The pixel values of the largest population were the measured light intensities.

To compare the light intensity measured with the absorbance measured by a spectrophotometer, conversion of the intensity of light to optical density (O.D.) was required by eq 1

1 $$O.D.=-\log\left(\frac{I_{o}}{I_{m}}\right)$$

since (*I*_0_) corresponds to the intensity of the blank signal and (*I*_m_) corresponds to the intensity of the measurement.^37^ A method using direct coupling of a smartphone to the 3D printed GDME device and the spectrophotometric method was evaluated following the recommendations of the National Health Surveillance Agency (Anvisa).^38^ The lowest concentration was the concentration that generated a 15% deviation in the measurements. For each of the analytical curves in milk, ANOVA (Analysis of Variance) regression tests were performed to verify whether the data were adjusted for both smartphone image detection and spectrophotometric detection. For this, the tabulated *F* values were compared with the critical *F* value at the 95% confidence level. The formaldehyde concentration in the straightening cream sample was calculated for each absorbance and integration area measurement. The sample standard deviation of the concentrations was used to estimate the method precision in triplicate.

### 2.6 Preparation for Analytical Samples

#### 2.6.1 Milk Samples

##### 2.6.1.1 Smartphone Detection

Analytical curves were prepared using different types of milk (whole, semi-skim, skim, lactose-free, pasteurized). For that, the procedure followed the schematic in Figure 2b. 10 mL of milk was added to the lower part of the GDME and spiked with formaldehyde solution (between 5.00 and 50.0 μL of a 5000 mg L^–1^ standard solution), followed by the addition of 50.0 mg of NaCl, and 100 μL of HCl 6 mol L^–1^. The GDME was then closed with the 3D-printed lid, and two perpendicular pieces of PTFE tape were placed over the hole in the lid. To fix the PTFE tape in place, the 3D-printed square with a center hole was placed over it. The last step in preparing the GDME for extraction was to add the derivatizing solution (500 μL) to the PTFE tape.

The extraction was performed by placing the GDMEs in a heated bath at 70 °C for 50 min. After the extraction period, the GDMEs were removed and allowed to rest for 10 min before smartphone detection. For that, the smartphone support (Figure 3) was placed over the GDME, and the digital image was collected.

##### 2.6.1.2 Spectrophotometric Detection

To compare the smartphone data, the spectrophotometric method was performed. After the digital image was collected, the derivatizing solution was transferred to a microvolume cuvette, and the absorbance was registered at 412 nm.

#### 2.6.2 Straightening Hair Cream

##### 2.6.2.1 Smartphone and Spectrophotometric Detection

Different samples of straightening hair cream were tested for formaldehyde. To do this, approximately 1.00 g of the sample was weighed directly into the GDME device. Extraction was allowed to occur for 10 min at 70 °C using PTFE tape and 500 μL of acetylacetone solution. After a positive formaldehyde result in the sample, formaldehyde extraction from straightening hair cream was optimized using a Box-Behnken design with 0.1 g of sample at 50 °C, with time varying from 5 to 15 min. The salting-out effect with NaCl was performed at 15–25% (w/v), and the pH was adjusted with a 6 mol L^–1^ HCl solution to between 50 and 100 μL. The GDME device was prepared with PTFE tape and 500 μL of acetylacetone solution. The solutions were left to stand for 10 min before absorbance was measured.

Although NaCl and HCl were evaluated during optimization, the final optimized method for the straightening hair cream was performed without salt and acid addition, as it did not significantly improve extraction efficiency. Under optimized conditions (20 min at 50 °C, without salt or acid), the formaldehyde content of the straightening hair cream sample was determined using the standard addition method. Increasing volumes (100–300 μL) of a 1000 mg L^–1^ formaldehyde solution were added. Water was also added to all GDME devices to keep the headspace volume constant. After the extraction period, digital images and absorbance readings were made.

##### 2.6.2.2 Chromatographic Method

To compare the measured values with another analytical approach, an analytical curve was generated using the standard addition method with headspace gas chromatography–mass spectrometry (HS-GC-MS). A 2050 mg sample of the straightening hair cream was weighed and diluted to 25.0 mL with deionized water. A 1.00 mL aliquot of the prepared solution was separated, and formaldehyde standard (1000 mg L^–1^) was added in different volumes (20–150 μL). Water was added to ensure the final volume of all vials was the same. Also, 0.20 g of NaCl was added to the headspace vial.

The conditions of the HS-GC-MS analysis were as follows: column oven temperature 35 °C for 3 min, then 25 °C*/*min until 200 °C for 2.4 min; split ratio 10:1; column Zebron ZB-WAX 0.25 μm, 15 m; injection temperature 200 °C; pressure 14.0 kPa; total flow 16.2 mL min^*–*1^; column flow 1.20 mL min^*–*1^; injection volume 500 μL. Auto sampler parameters: incubation temperature 60 °C; incubation time 15 min; syringe temperature 60 °C; agitator speed 250 rpm; prepurge time 5 s; injection flow rate 10 mL min^*–*1^; postpurge time 10 s. Data acquisition parameters: ion source temperature 200 °C; interface temp 200 °C; solvent cut time 1.65 min; sim mode *m*/*z* 29.00 and 30.00.

## 3 Results and Discussion

### 3.1 Manufacture and Evaluation of 3D Printed GDME Devices

PDMS was applied to the inside of the GDME base and the inside of the lid, as shown in Figure 5a. Figure 5b shows the closed GDME device. After assembly, the GDME device could be used to extract formaldehyde in the heated bath.

**Figure 5:**
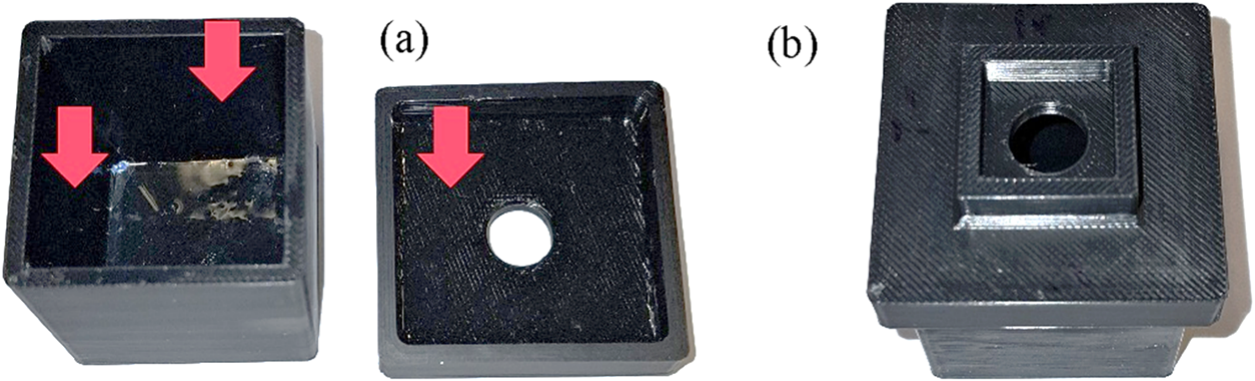
Photographs of the final 3D-printed devices and assembly steps: (a) closed GDME device; (b) 3D-printed GDME prototype, where red arrows indicate the specific locations for PDMS application.

Results from the GDME devices are presented in Figure S2, which was performed in triplicate, and the error bars represent RSD values. The best extraction results were obtained with prototype 3. With a height of 50 mm and a volume of 6.00 × 10^3^ mm^3^, it exhibited a higher average absorbance (0.892 *±* 0.087) than prototypes 1 to 5. However, prototype 2 achieved a formaldehyde extraction (0.860 *±* 0.048), 4% less than prototype 3, with a smaller error. Prototype 6 was built by increasing the headspace volume of prototype 2 and showed better extraction, but because the measurement deviations were high, prototype 2 was chosen for analyses of milk and cosmetic samples.

### 3.2 Analysis of Milk

Analytical curves (Figure S3) were obtained by adding different volumes of a standard formaldehyde solution. The regression models were obtained using simple linear regression. For the smartphone, the correlation coefficient was above 0.95, whereas for the spectrophotometric approach, it was above 0.98. The limit of quantification was set at the concentration at which the RSD reached 15%, which was 2.50 mg L^–1^.

Figure 6 shows the correlation between the concentrations calculated using the proposed smartphone-based analysis and those obtained by the reference spectrophotometric method. The linear regressions for all sample sets were consistent (see Supporting Information for the regression equations), with slopes approximating 1.0 (unity). These results indicate a strong agreement between the methods and demonstrate that the proposed approach is free from systematic bias.

**Figure 6:**
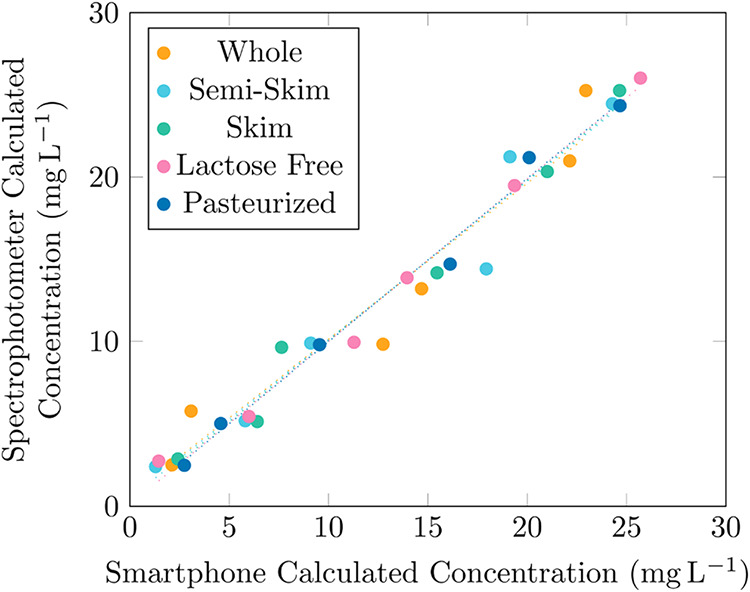
Correlation chart of the proposed and reference methods for lactose-free and pasteurized milk.

In the determination of formaldehyde in different milk samples, according to the figures of merit presented, the analytical curves for the different types of milk were similar (Table 1). From the extraction data, whole milk showed the highest sensitivity, followed by lactose-free milk and semi-skim milk. When analyzing the nutritional information on the milks used, the carbohydrate, protein, and calcium content were the same across all milk samples. However, the fat content decreases as a whole, in semi-skim, and skim milk. The lactose-free milk examined in this study had a fat content close to that of semi-skim milk, but with twice the sodium content. It is believed that the fat and sodium content positively influenced formaldehyde extraction. Since formaldehyde forms hydrogen bonding with water, the addition of an electrolyte causes hydrogen bonding interactions between formaldehyde and water to weaken.^39^ At low concentrations, the RSD limited the method’s LOQ to 2.5 mg L^–1^, and at the highest concentrations in the analytical curve, the RSD reached a minimum of 0.594%.

**Table 1:** Analytical Figures of Merit from Calibration Curve Data for Different Types of Milk Extracted by 3D Printed GDME Devices

Milk type	Regression Equation	Concentration	LOQ	RSD (%)
Whole	O.D. = 0.0182[HCHO] + 0.0487	2.50 – 25.0 mg L^–1^	2.50 mg L^–1^	0.549– 9.88
Semi-skim	O.D. = 0.0138[HCHO] + 0.0280			5.81–15.0
Skim	O.D. = 0.0124[HCHO] + 0.0677			6.02–15.0
Lactose-free	O.D. = 0.0157[HCHO] + 0.0309			1.72–13.7
Pasteurized	O.D. = 0.0128[HCHO] + 0.0729			4.30–14.4

In all regression tests, ANOVA was performed using spectrophotometer data and smartphone images; none showed a lack of fit. To prove that both methods yielded equivalent results, an F-test was performed. The variable analyzed for this response was the *p*-value, which was greater than 0.05 (95% confidence level), indicating that the variables are equivalent; otherwise. With this result, it was possible to perform the *t* test, assuming that the variables were equivalent or not, based on the F-test. The *p*-value was reanalyzed, yielding a value above 0.05; therefore, both systems evaluated are considered equivalent. All underlying statistical parameters are available for download from the GitHub repository linked in Supporting Information.

For UHT (ultrahigh-temperature processing) whole milk, semi-skim milk, and lactose-free milk, the F-test indicated that the variables were equivalent, with a *p*-value greater than α = 0.05. For pasteurized whole milk and skim milk, the variables differed (*p*-value less than α = 0.05), indicating an initial difference between the variables obtained using the two methods (alternative hypothesis). To verify rejection of the null hypothesis, the *t* test was performed. However, with the *t* test, all milk samples showed two-tailed *p*-values greater than 0.05, indicating that the data obtained by spectrophotometry and smartphone digital images are, in fact, equivalent. Figure 6 supports the correlation tests between smartphone and spectrophotometer data. Compared with other publications that reported formaldehyde detection in milk samples and the AOAC method, Temel achieved an LOQ of 1.67 μg L^–1^ using a large volume of organic solvent.^40^ Costa et al.^41^ reported a linear range between 2.00 and 20.0 μg L^–1^ using an online sample preparation system. This work achieved a higher LOQ, using a simpler method that can be performed by a greater number of users, with safer and greener reagents.

### 3.3 Analysis of Straightening Hair Cream

A preliminary analysis to detect formaldehyde in the straightening hair cream revealed that 1.00 g of the sample and an extraction time of 10 min at 70 °C were sufficient to exceed the spectrophotometer’s working range. The higher formaldehyde concentration in the straightening hair cream samples compared to the milk samples necessitated a lower heating temperature to prevent the absorbance of the derivatizing solution from exceeding the spectrophotometer’s upper limit. By maintaining a temperature of 70 °C and reducing the time, the deviation in the absorbance measurements was found to be high. Lower temperatures and longer extraction times yielded more accurate results (lower RSD) for both the smartphone and spectrophotometric analysis. Therefore, to optimize the extraction conditions (extraction time, NaCl, and HCl amount) for formaldehyde in the straightening hair cream, the sample amount was reduced to 0.1 g, and the temperature was set to 50 °C.

Analyzing experimental data from the Box-Behnken design, the Pareto chart in Figure 7a indicates that time was the only significant factor in the extraction process, with longer times yielding higher extraction efficiency and, consequently, higher absorbance. This was expected, as longer extraction times allow more formaldehyde to be volatilized and to react with the derivatizing solution.

**Figure 7:**
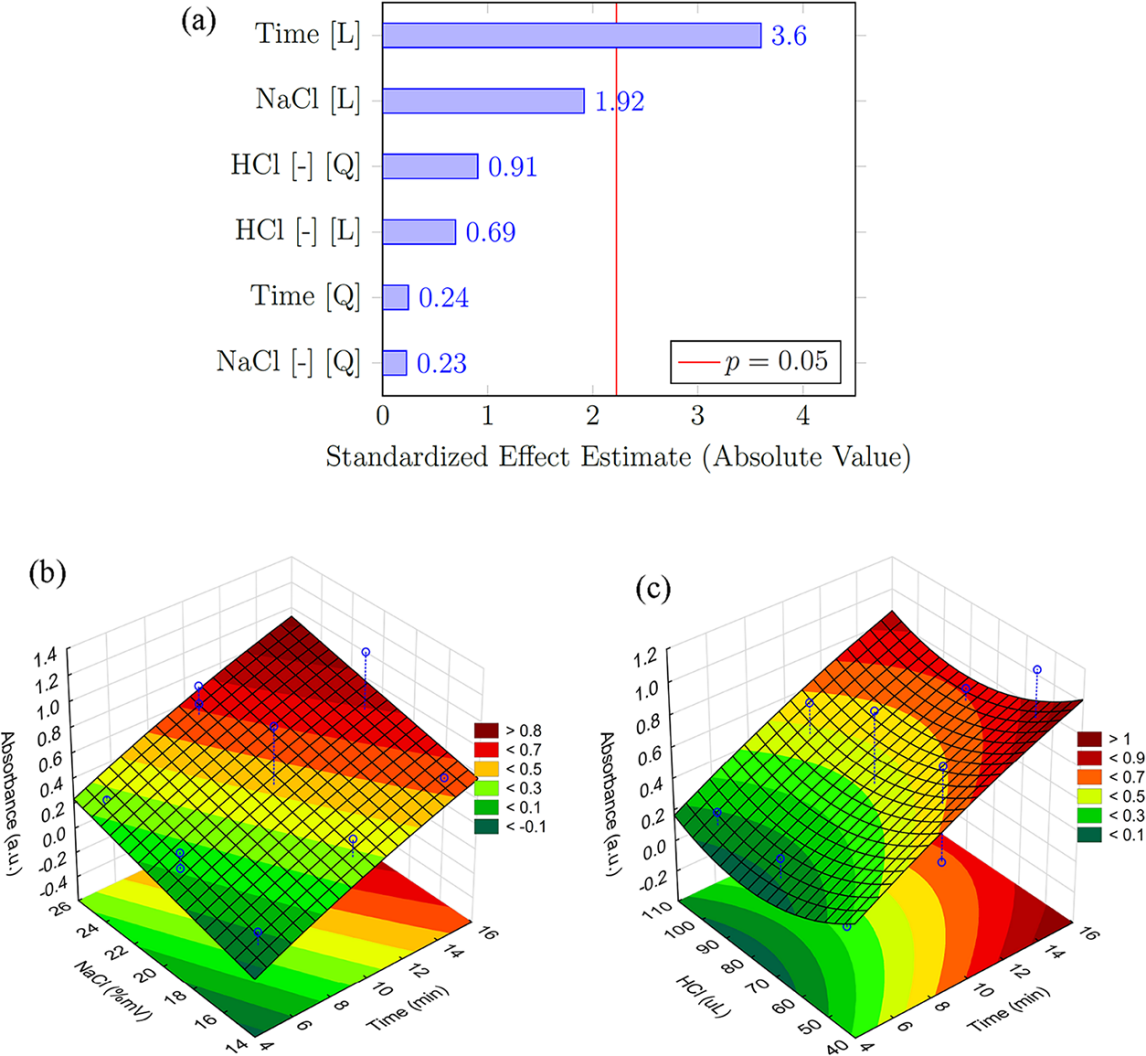
(a) Pareto chart of Box-Behnken design for optimizing formaldehyde extraction in a straightening hair cream sample. Response surface optimization of formaldehyde extraction from straightening hair cream. (b) Effect of NaCl concentration and extraction time. (c) Effect of HCl concentration and extraction time.

Figure 7b shows the influence of NaCl content and time for the extraction of formaldehyde. NaCl facilitates the volatilization of formaldehyde; however, based on the level curves, NaCl has a small influence on formaldehyde extraction and can be substituted with a small increase in time, improving the green metrics of the proposed method. The use of HCl presented in Figure 7c showed a negative influence and was not used for method calibration. However, the worsening of the extraction upon the addition of HCl can be explained by the water added to the sample, which was sufficient to form hydrogen bonds with the formaldehyde. Based on the results, the optimal condition for removing formaldehyde from the cream was determined to be 20 min at 50 °C, without the addition of salt or acid.

Under the conditions established above, an analytical curve was produced by standard addition. Figure 8a compares the absorbance values from smartphone images with those recorded by the spectrophotometer. For digital images, the linear regression equation was O.D. = 1.06 [HCHO] + 0.153, with an *R* ^2^ value of 0.981. For the spectrophotometric approach, the linear regression equation was *A* = 2.26­[HCHO] + 0.338 with an *R* ^2^ value of 0.969. The formaldehyde concentration calculated from the smartphone images was 0.144 *±* 0.009% (w/w), and that from the spectrophotometer was 0.149 *±* 0.012% (w/w). The accuracy of the smartphone analysis using the GDME device was 96.6%, compared with the spectrophotometer.

**Figure 8:**
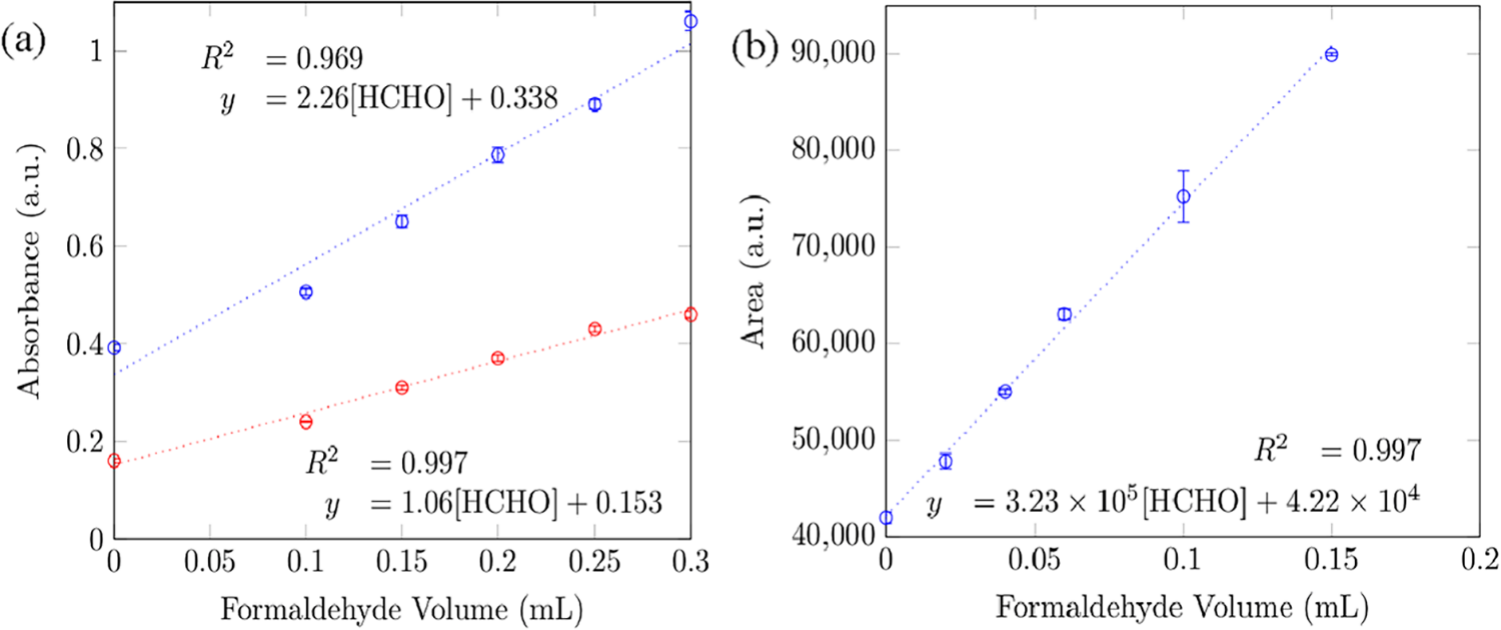
(a) Analytical curve obtained by standard addition to straightening hair cream using spectrophotometry (blue) and smartphone (red). (b) Analytical curve obtained by standard addition in straightening hair cream by HS-GC-MS.

A difference in sensitivity between smartphone and spectrophotometric detection was expected. Spectrophotometers have more sophisticated optical components and detectors, as well as electronic circuits specifically designed for this purpose, whereas smartphones are designed to generate images from camera-recorded signals.

The analytical performance of the proposed 3D-printed GDME was compared with recently reported methods and established protocols for formaldehyde determination, as summarized in Table 2. A significant benchmark is the work of Deniz & Aydin,^42^ who achieved a slightly lower LOQ of 0.089 mg L^–1^ using H-acid in an aqueous medium. However, their method relies on direct mixing solutions, which, while sensitive, are primarily suitable for clear aqueous samples and require conventional spectrophotometric instrumentation. In contrast, our method provides a critical advantage: matrix isolation. By employing a 3D-printed GDME architecture, the analyte is separated from the complex matrices of milk and cosmetics via gaseous diffusion. This step eliminates interferences from nonvolatile components, proteins, and fats that would otherwise compromise direct optical measurements.

**Table 2:** Comparative Performance of the Proposed 3D-Printed GDME with Other Reported Methods for Formaldehyde Determination

Parameter	Brandão (2018)^26^	Deniz & Aydin (2026)^42^	This Work
Reagent	Acetylacetone (Hantzsch)	H-acid (Sodium salt)	Acetylacetone (Hantzsch)
Matrix	Cosmetic and hygiene products	Aqueous medium	Milk and cosmetics
Preparation	Gas Diffusion Microextraction (GDME)	Direct mixture in solution	Gas Diffusion Microextraction (GDME)
LOQ	6.60 mg L^–1^	0.089 mg L^–1^	0.132 mg L^–1^
Selectivity	Very High (GDME + Chemical Selectivity)	High (tested with other aldehydes)	Very High (GDME+ Chemical Selectivity)
Instrumentation	Spectrophotometric	Spectrophotometric	Smartphone

Furthermore, while the protocol by Brandão et al.^26^ also, utilizes GDME, our design significantly improved sensitivity, reducing the LOQ from 6.60 to 0.132 mg L^–1^ (calculated for straightening hair cream) and drastically lowering the required sample amount to only 0.10 g. Finally, the integration of smartphone-based detection and MATLAB automation shifts the analysis from the laboratory bench to a portable and cost-effective format. This synergy between additive manufacturing and digital image colorimetry demonstrates that high analytical rigor can be maintained without the need for expensive high-mass PTFE components or centralized laboratory equipment.

Reusability can be achieved by washing the GDMEs devices with water and ethanol. After use, the GDMEs were cleaned and evaluated for residual contamination. Three GDME devices were randomly selected to repeat the same method used for formaldehyde extraction of milk samples (a heated bath at 70 °C for 50 min and 500 μL of acetylacetone solution) to evaluate cross-contamination or carryover. After the extraction period, the absorbance of the derivatizing solution was measured using a spectrophotometer. The readings showed no difference compared to the blank.

As a comparison method, formaldehyde in the straightening hair cream was determined by HS-GC-MS. The standard addition calibration method presented in Figure 8b provided a linear regression equation of Area = 3.23 × 10^5^ [HCHO] + 4.22 × 10^4^ and *R* ^2^ = 0.997. The calculated formaldehyde concentration was 0.160 ± 0.009%. When comparing the accuracy of GDME-smartphone analysis with HS-GC-MS, the accuracy was 90%.

### 3.4 Cost Analysis

The production cost of the printed GDME device was USD 7.0, including ABS filament and PDMS. The cost of milk analysis was USD 0.45, while the straightening hair cream analysis was USD 0.28 per sample. The estimated cost of the HS-GC-MS approach was USD 10.0 per sample. In laboratories that routinely perform milk and cosmetics analysis, the cost of the 3D-printed GDME device with smartphone detection is significantly reduced by its reusability and the ability to customize its shape and size for different samples. When comparing the different techniques, the chromatographic approach has greater sensitivity; however, it incurs higher analysis costs. Despite its lower sensitivity, the 3D-printed GDME smartphone approach detected formaldehyde in a straightening hair cream sample at a significantly lower analysis cost.

### 3.5 Greenness Evaluation

To evaluate the green metrics of the method,^43^ the AGREE (Analytical GREEnness Metric Approach) and the software platform were used and compared with the official methods^44^ The platform considers the entire analytical process to assign a score between 0 and 1 to the evaluated procedure. Using the AGREE methodology to evaluate green metrics, the proposed method for milk samples received a score of 0.61, while the official AOAC 931.08 method received a score of 0.34. In the straightening hair cream samples, the proposed method received a score of 0.75, while the official method received a score of 0.48.^44^ The practicability of the proposed method was assessed using the BAGI algorithm, resulting in a score of 75.0. This represents a significant improvement over the official method, which received a score of 57.5.

This study demonstrates the potential of 3D-printed GDME devices for quantifying formaldehyde in milk and hair straightening cream samples, highlighting their low cost and single-step, simultaneous sample preparation and quantification. It also demonstrates the simultaneous preparation of multiple samples and analysis using simple analytical techniques, with portable instrumentation and smartphone-based detection. Furthermore, an image processing algorithm was developed in MATLAB that involves processing, filtering, blue-matrix selection, and histogram generation to map the pixel distribution. The quantification analysis was based on the light intensity at the pixel with the highest population, converted to optical density. The development of this algorithm enabled the rapid, simultaneous analysis of images, enabling efficient processing of large volumes of photographs and minimizing errors from brightness adjustment and white balance, which can compromise the accuracy and reproducibility of results.

No interference was observed in 3D-printed GDME during the measurements of milk and straightening hair cream, even considering the complex matrix of the samples. The device can be reused after thorough cleaning, demonstrating that the system is suitable for large-scale manufacturing, reducing per-unit cost, and making it an efficient tool for scientific and analytical applications. The proposed methodology demonstrated strong environmental performance (AGREE and BAGI), efficient use of resources, minimal generation of toxic waste, and minimal sample preparation. In addition, it supports up to 12 simultaneous analyses per hour, at a lower cost per analysis than robust techniques such as HS-GC-MS. This ensures that the method is sustainable and exhibits excellent ecological performance.

## 4 Conclusion

This study demonstrated the feasibility of using 3D printing to produce GDME devices for the determination of formaldehyde in milk and cosmetics samples. The MATLAB-based automation for analyzing RGB data from digital images reduced the time required to retrieve RGB values and provided an efficient, practical method that was statistically equivalent to analyses using a spectrophotometer and HS-GC-MS. The results demonstrate that the GDME design and headspace volume affect extraction efficiency and analytical precision when using the 3D-printed GDME devices. A comparison of HS-GC-MS results showed that the printed GDMEs can perform quantitative chemical analyses at a fraction of the initial and maintenance costs of the HS-GC-MS approach. An evaluation of green notes revealed that the proposed method is greener than official methods. 3D-printed GDME devices are easily customized, and their small size allows their use in portable methods. By coupling smartphone detection with 3D-printed GDME devices, it was possible to extract and quantify formaldehyde in milk and straightening hair cream samples at a lower initial investment, thereby reducing the cost per analysis by 95% compared to HS-GC-MS, while achieving the same results, as confirmed by statistical evaluation. Regarding future improvements, optimizing printing parameters or using resin-based 3D printers (SLA/DLP) could enhance the sealing properties of GDME housing, potentially eliminating the need for PDMS coating. This would further decrease production costs and complexity, allowing the devices to be ready for immediate use upon fabrication.

## Supplementary Material



## Data Availability

Data used is available throughout the manuscript text.

## Notes

Illustration of all GDME prototypes evaluated in this study; performance study for each GDME prototype; analytical curves for each milk sample, in Figures S1 to S3; experimental conditions for the optimization experiments; ANOVA table for the optimization experiments in Tables S1 and S2; additionally, a GitHub repository link containing all analytical and statistical data, as well as the 3D models used for formaldehyde determination (PDF)
