# Supplementary material for: Smartphone Determination of Formaldehyde in Milk and Cosmetics Samples Using Gas-Diffusion Microextraction Devices Prepared by 3D Printing
Source: ACS Omega. 2026 Jul 2;11(27):40617–27. doi: 10.1021/acsomega.6c03601 (PMC13382816; doi:10.1021/acsomega.6c03601)
Supplement: Supplementary file 1 [file ao6c03601_si_001.pdf]

# Smartphone determination of formaldehyde in milk and cosmetics samples using gas-diffusion microextraction devices prepared by 3D printing

Juliana Casanova Pinho[1], Gabriel Baroffaldi Piassalonga[1], Saidy Cristina Ayala-Durán[1], João Pedro Silva[1], Josias Merib[2], Jared L. Anderson[3], Maria Valnice Boldrin Zanoni[1], and Paulo Clairmont Feitosa de Lima Gomes[1*]

[1] Sao Paulo State University (UNESP), Institute of Chemistry, Department of Analytical Chemistry, Physical Chemistry and Inorganic Chemistry, National Institute for Alternative Technologies of Detection, Toxicological Evaluation and Removal of Micropollutants and Radioactives (INCT-DATREM), São Paulo State University (UNESP), Araraquara, São Paulo 14800-060, Brazil.

[2] Universidade Federal de Ciências da Saúde de Porto Alegre, Porto Alegre, Rio Grande do Sul, Brasil; Programa de Pós-graduação em Biociências e Departamento de Farmacociências, Porto Alegre, Rio Grande do Sul 90050-170, Brasil.

[3] Department of Chemistry, Iowa State University, Ames, IA 50011, United States.

**Corresponding author:** Paulo Clairmont Feitosa de Lima Gomes

**Address:** Rua Prof. Francisco Degni 55, Araraquara, SP, 14800-060, Brazil

**Phone:** +55 (16) 3373 9613

**e-mail**: paulo.clairmont@unesp.br

# Appendix A

3D printing models, milk extraction data, and statistical treatments can be downloaded from GitHub via the link:

https://github.com/gabriel-baroffaldi/Supplementary-Information---Smartphone-determination-of-formaldehyde

**Table S1**: Dimensions of the different 3D printed GDMEs to evaluate the best model for formaldehyde extraction.

| Prototype | Height (mm) | Volume ($mm^3$) |
|---|---|---|
| 1 | 30.0 | $6.00 \times 10^3$ |
| 2 | 40.0 | $6.00 \times 10^3$ |
| 3 | 50.0 | $6.00 \times 10^3$ |
| 4 | 90.0 | $6.00 \times 10^3$ |
| 5 | 120.0 | $6.00 \times 10^3$ |
| 6 | 40.0 | $12.0 \times 10^3$ |

**Figure S1:** Technical layouts of the six 3D-printed GDME prototypes investigated for optimization of the extraction process (Section 3.1)

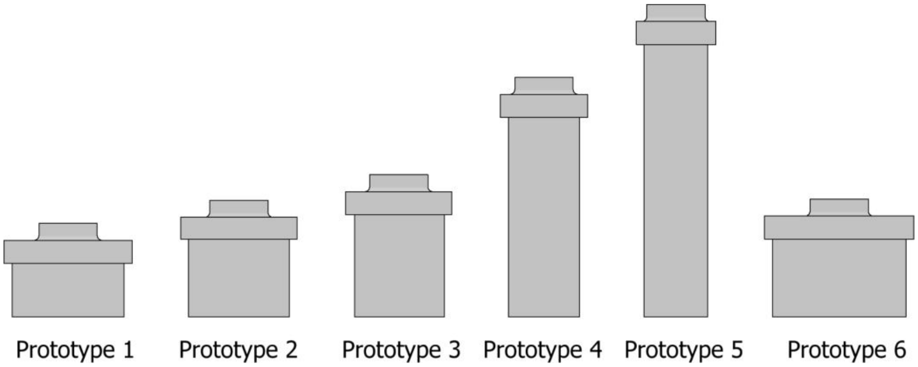

**Figure S2:** Column chart showing the average absorption of each 3D printed GDME prototype evaluated in an aqueous medium with 25 mg $L^{-1}$ of formaldehyde.

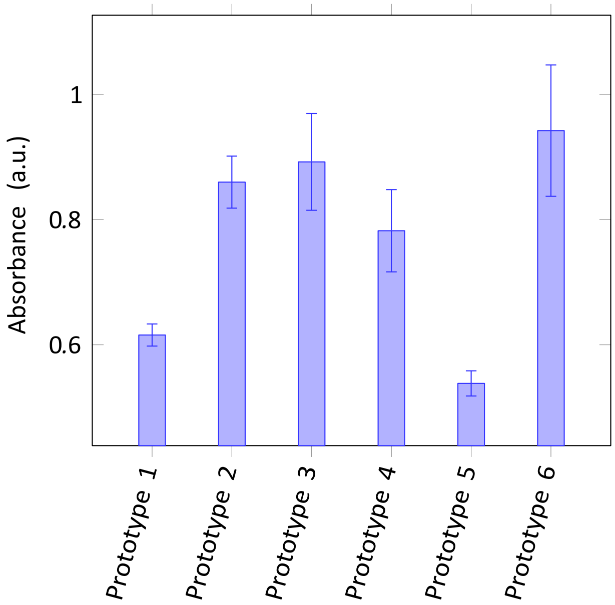

**Figure S3:** Smartphone analytical curve for the different types of milk. (a) Analytical curve for whole and semi-skim milk. (b) Analytical curve for skim and lactose-free milk. (c) Analytical curve for pasteurized milk.

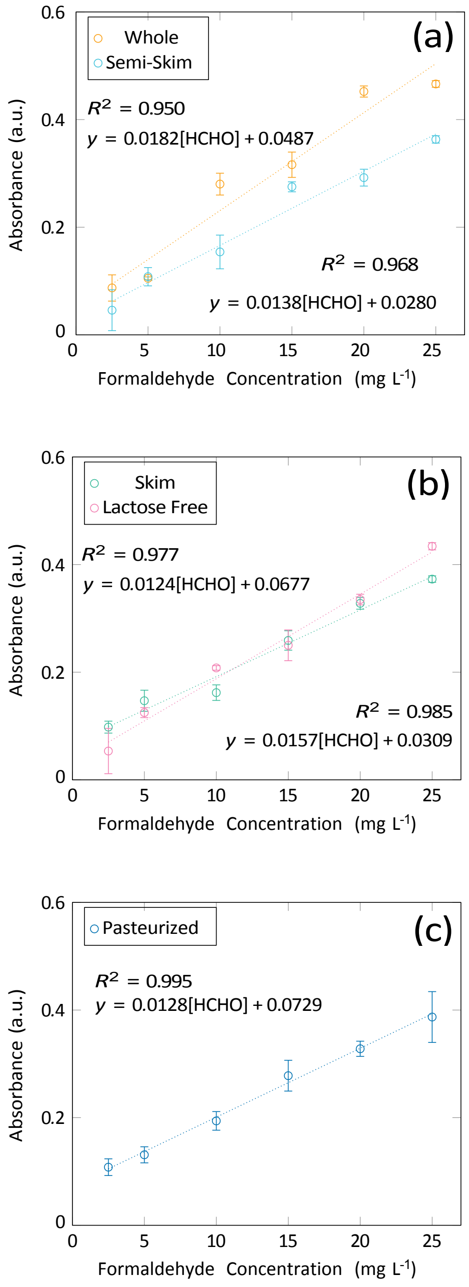

**Table S2:** Conditions of the experiments carried out on the Box-Behnken planning.

| Time (min) | NaCl (%w/v ) | HCl (μL) | Absorbance |
|---|---|---|---|
| 5.00 | 15.0 | 75.0 | 0.0630 |
| 10.0 | 25.0 | 50.0 | 0.817 |
| 10.0 | 15.0 | 100 | 0.422 |
| 10.0 | 15.0 | 50.0 | 0.225 |
| 10.0 | 20.0 | 75.0 | 0.388 |
| 10.0 | 20.0 | 75.0 | 0.886 |
| 10.0 | 20.0 | 75.0 | 0.203 |
| 15.0 | 25.0 | 75.0 | 0.771 |
| 15.0 | 20.0 | 100 | 0.518 |
| 5.00 | 20.0 | 100 | 0.241 |
| 5.00 | 20.0 | 50.0 | 0.117 |
| 10.0 | 20.0 | 75.0 | 0.350 |
| 15.0 | 20.0 | 50.0 | 1.14 |
| 10.0 | 20.0 | 75.0 | 0.329 |
| 15.0 | 15.0 | 75.0 | 0.553 |
| 5.00 | 25.0 | 75.0 | 0.239 |
| 10.0 | 25.0 | 100 | 0.674 |

**Table S3:** Anova table from Box-Behnken experiments.

| | SS | df | MS | F | p |
|---|---|---|---|---|---|
| (1)Time (min) L+Q | 0.678824 | 2 | 0.339412 | 6.513378 | 0.015447 |
| (2)NaCl (%mV) L+Q | 0.191607 | 2 | 0.095804 | 1.838490 | 0.208953 |
| (3)HCl (uL) L+Q | 0.067737 | 2 | 0.033868 | 0.649938 | 0.542790 |
| Error | 0.521100 | 10 | 0.052110 | | |
| Total SS | 1.458334 | 16 | | | |

ANOVA; Var.:Absorbance; R-sqr=.64267; Adj:.42828

3 3-level factors, 1 Blocks, 17 Runs; MS Residual=.05211 DV: Absorbance
